# Ten-Year Disease Control of Metastatic Pancreatic Neuroendocrine Tumour Treated With Everolimus

**DOI:** 10.7759/cureus.112071

**Published:** 2026-07-05

**Authors:** Madalena Machete, Bruno M Silva, Pedro Simões, Luísa Leal-Costa, José Alberto Teixeira

**Affiliations:** 1 Oncology, Hospital Beatriz Ângelo, Loures, PRT

**Keywords:** everolimus, gastrointestinal neuroendocrine neoplasms, mtor inhibitors, multidisciplinary team, pancreatic neuroendocrine tumour

## Abstract

Management of metastatic pancreatic neuroendocrine tumours remains challenging due to clinical heterogeneity and limited high-quality evidence, especially regarding treatment beyond first-line therapy. Optimal treatment strategies in the second-line setting are not well established.

We report the case of a woman in her late 30s who presented with progressively debilitating back pain and was diagnosed with an intermediate-grade pancreatic neuroendocrine tumour with bone and liver metastases. Based on negative findings on somatostatin receptor-based imaging, she commenced first-line chemotherapy with capecitabine and temozolomide. In parallel, local therapy for bone metastases was administered through radiotherapy and surgical intervention.

After eight months of therapy, there was significant disease progression, with numerous bone and lymph node metastases. At this point, treatment was switched to everolimus as second-line therapy. The patient experienced rapid functional improvement, a marked reduction in tumour burden, and only manageable side effects (stomatitis and hypertriglyceridaemia). Over time, she achieved sustained disease control with everolimus.

This case illustrates a durable clinical response to second-line everolimus in a patient with metastatic pancreatic neuroendocrine tumour and adds to the growing clinical experience with this treatment. It also highlights the benefits of a multidisciplinary approach to the management of metastatic disease.

## Introduction

Pancreatic neuroendocrine tumours (NETs) are rare and account for less than two per cent of all pancreatic cancers [1]. These well-differentiated neoplasms arise from pancreatic islet cells and are classified according to their proliferation rate into low-, intermediate-, and high-grade tumours [1,2]. Most pancreatic NETs are non-functional, and their clinical presentation depends on both the extent of the primary tumour and the presence of distant metastases [3].

Pancreatic NETs typically overexpress somatostatin receptors (SSRs) on the tumour cell surface, which can be detected using SSR-based functional imaging. SSR negativity on SSR-directed imaging studies is associated with less differentiated NETs, more aggressive biological behaviour, and limited eligibility for SSR-targeted therapies [4].

Pancreatic NETs have a worse prognosis than NETs arising from other primary sites [5]. In recent years, several therapeutic options have been approved for the treatment of metastatic pancreatic NETs (including somatostatin analogues, targeted therapies such as everolimus and sunitinib, peptide receptor radionuclide therapy, and chemotherapy), contributing to improved overall survival. However, evidence regarding the optimal sequencing of these treatments remains limited [6].

The mammalian target of rapamycin (mTOR) pathway plays a central role in the pathogenesis of NETs. It comprises two protein complexes, mTOR complex 1 (mTORC1) and mTOR complex 2 (mTORC2), which regulate cell proliferation and survival. Everolimus is an oral mTOR inhibitor that binds to FK506-binding protein 12 (FKBP12), resulting in inhibition of mTORC1 signalling [6].

We report the case of a young patient with metastatic pancreatic NET who experienced rapid disease progression during first-line chemotherapy and subsequently achieved durable disease control with everolimus.

## Case presentation

A woman in her late 30s presented with a two-year history of progressively debilitating lower back pain with no accompanying symptoms. After several visits to the emergency room and to her attending physician, she was treated with trials of different analgesic and anti-inflammatory drugs, without improvement.

This patient had a history of papillary thyroid carcinoma (treated with total thyroidectomy and adjuvant radioactive iodine therapy) and Crohn's disease (previously treated with ileocolic resection for ileal stenosis). Past surgical history also included cholecystectomy and breast reduction surgery. Her regular medications included levothyroxine, prednisolone, mesalazine, duloxetine, and tramadol. There was no relevant family history.

She underwent computed tomography (CT) and magnetic resonance (MR) imaging of the spine for further investigation, and two osteolytic lesions in the L3 and L4 vertebrae were detected. Breast examination, mammography, and breast ultrasound were performed, with no suspicious findings. The pathology specimen from her previous breast surgery was reviewed and remained unremarkable.

**Figure 1:**
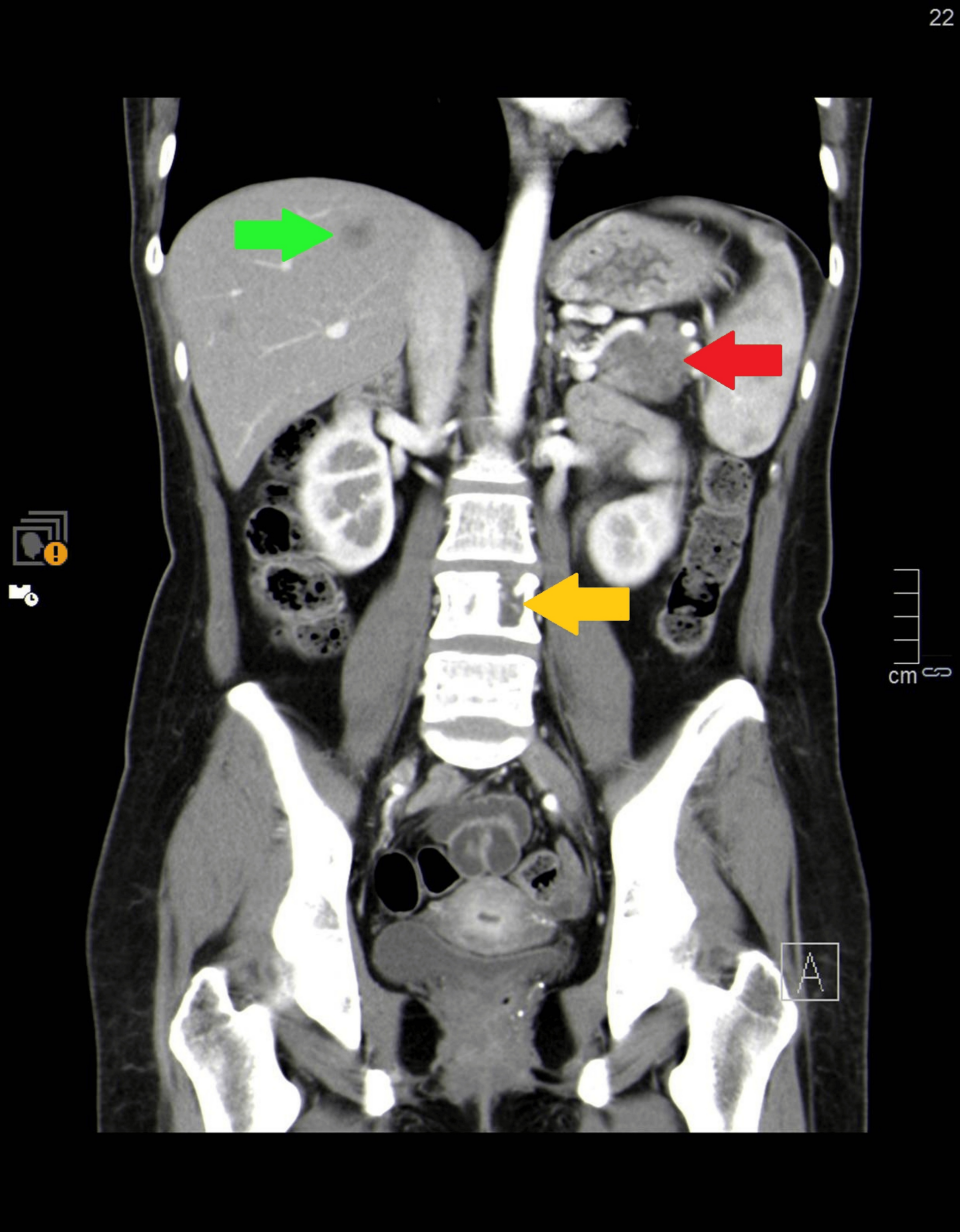
CT scan of the thorax, abdomen and pelvis showing one lesion in the distal pancreas (red arrow), one hepatic nodular lesion suggestive of metastasis (green arrow) and one osteolytic lesion in the L4 vertebral body (yellow arrow).

A CT scan of the thorax, abdomen, and pelvis (Figure 1) revealed a suspicious nodular lesion in the pancreatic tail, measuring approximately 28 × 30 mm, with slightly lobulated margins, hypodense on unenhanced imaging, and with reduced contrast enhancement. No calcifications within this lesion were identified, and no pancreatic duct dilatation was observed. In addition, 10 hepatic nodular lesions with similar imaging features were identified, raising a strong suspicion for metastatic disease. 

**Figure 2:**
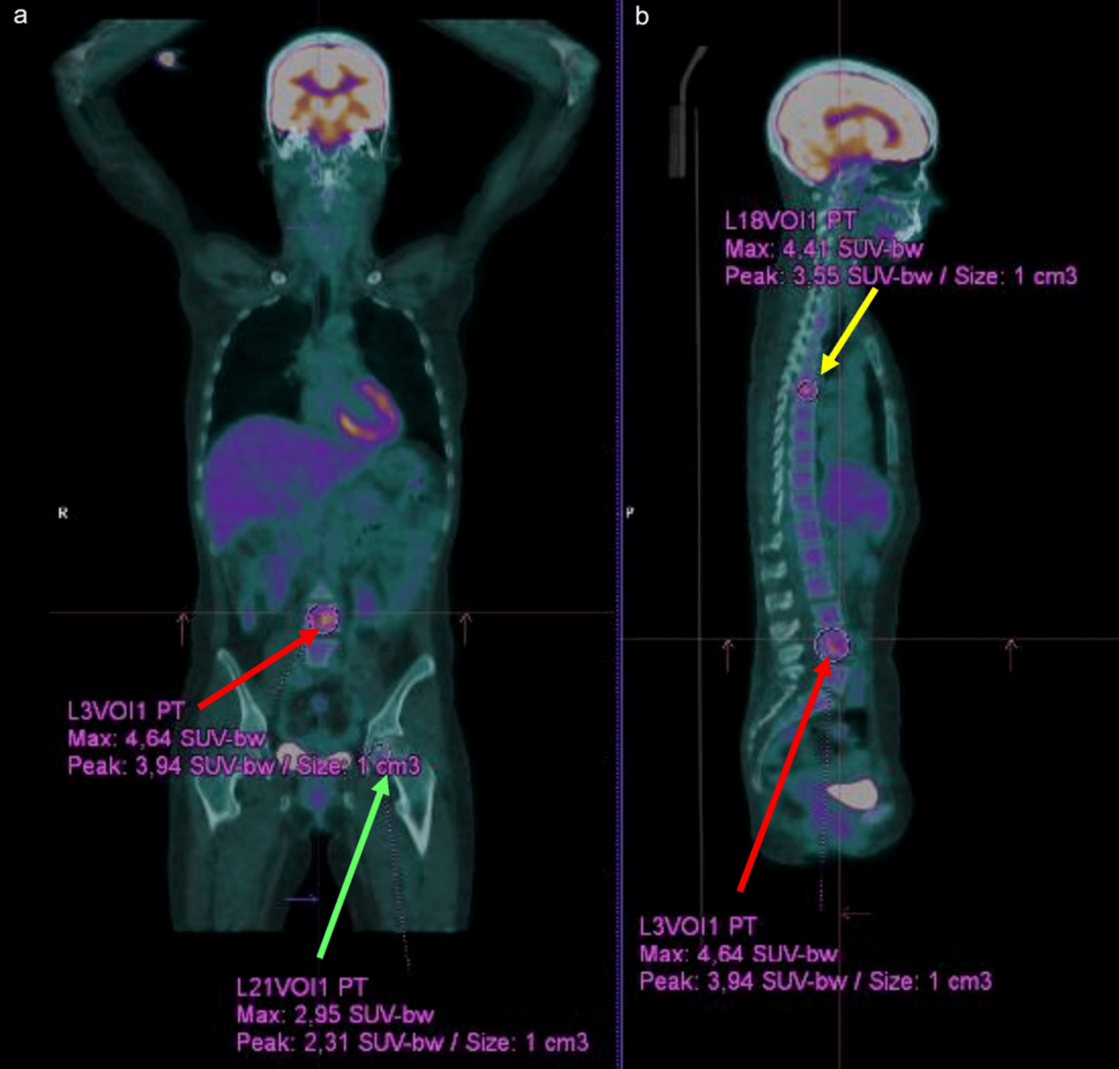
18F-FDG PET/CT with coronal (a) and sagittal (b) views demonstrating hypermetabolic osteolytic lesions involving the T6 (yellow arrow) and L4 vertebral bodies (red arrows), as well as in the left femoral head (green arrow), with SUVmax values ranging from 2.95 to 4.64. fluorodeoxyglucose, PET/CT: positron emission tomography/computed tomography, SUVmax: maximum standard uptake value.

Positron emission tomography with 2-deoxy-2-(fluorine-18)fluoro-D-glucose integrated with CT (^18^F-FDG PET/CT) showed moderate uptake in bone lesions involving the L3 and L4 vertebrae (the two lesions previously described), and additionally identified increased uptake in the T6 vertebra, two left costal arches and the left femoral head, with standard uptake values (SUV) between 2.95 and 4.64 (Figure 2). No increased uptake in the liver or pancreas was detected.

Upper and lower digestive endoscopies ruled out suspicious lesions in the gastrointestinal tract. The pathology from her previous bowel resection was also reviewed, with no new findings. Blood tests, including tumour markers, were within normal limits, with the exception of mild hypokalaemia, which was considered clinically insignificant (Table 1).

**Table 1:** Laboratory results at initial diagnostic investigation. AST: aspartate aminotransferase, ALT: alanine aminotransferase, ALP: alkaline phosphatase, GGT: gamma-glutamyl transferase, LDH: lactate dehydrogenase, CEA: carcinoembryonic antigen, NSE: neuron-specific enolase.

Laboratory tests	Values	Reference range
Haemoglobin	13.8	12.0-15.20 g/dL
White cell count	4.90	4.50-11.0 x 10^9^/L
Neutrophils	3.22	1.80-7.40 x 10^9^/L
Platelets	161	150-450 x 10^9^/L
Glucose	87	60-100 mg/dL
Urea	19	16.6-48.5 mg/dL
Creatinine	0.69	0.51-0.95 mg/dL
Total bilirubin	0.81	<1.20 mg/dL
AST	14	<32 UI/L
ALT	32	<33 UI/L
ALP	47	35-104 UI/L
GGT	36	<40 UI/L
LDH	157	135-214 UI/L
Sodium	142	136-145 mmol/L
Potassium	3.3	3.5-5.1 mmol/L
Chloride	100	98-107 mmol/L
Calcium	8.8	8.7-10.4 mmol/L
Magnesium	2.1	1.6-2.6 mg/dL
Albumin	4.3	3.5-5.2 g/dL
CEA	1.5	<3 ng/mL
CA 19.9	17.7	<37.0 U/mL
CA 15.3	4.9	<32.4 U/mL
CA 125	5.9	<30.2 U/mL
NSE	12.9	<18.3 ng/mL
Thyroglobulin	<0.2	<55.0 ng/mL
Chromogranin A	<5.00	<100.00 ng/mL

**Figure 3:**
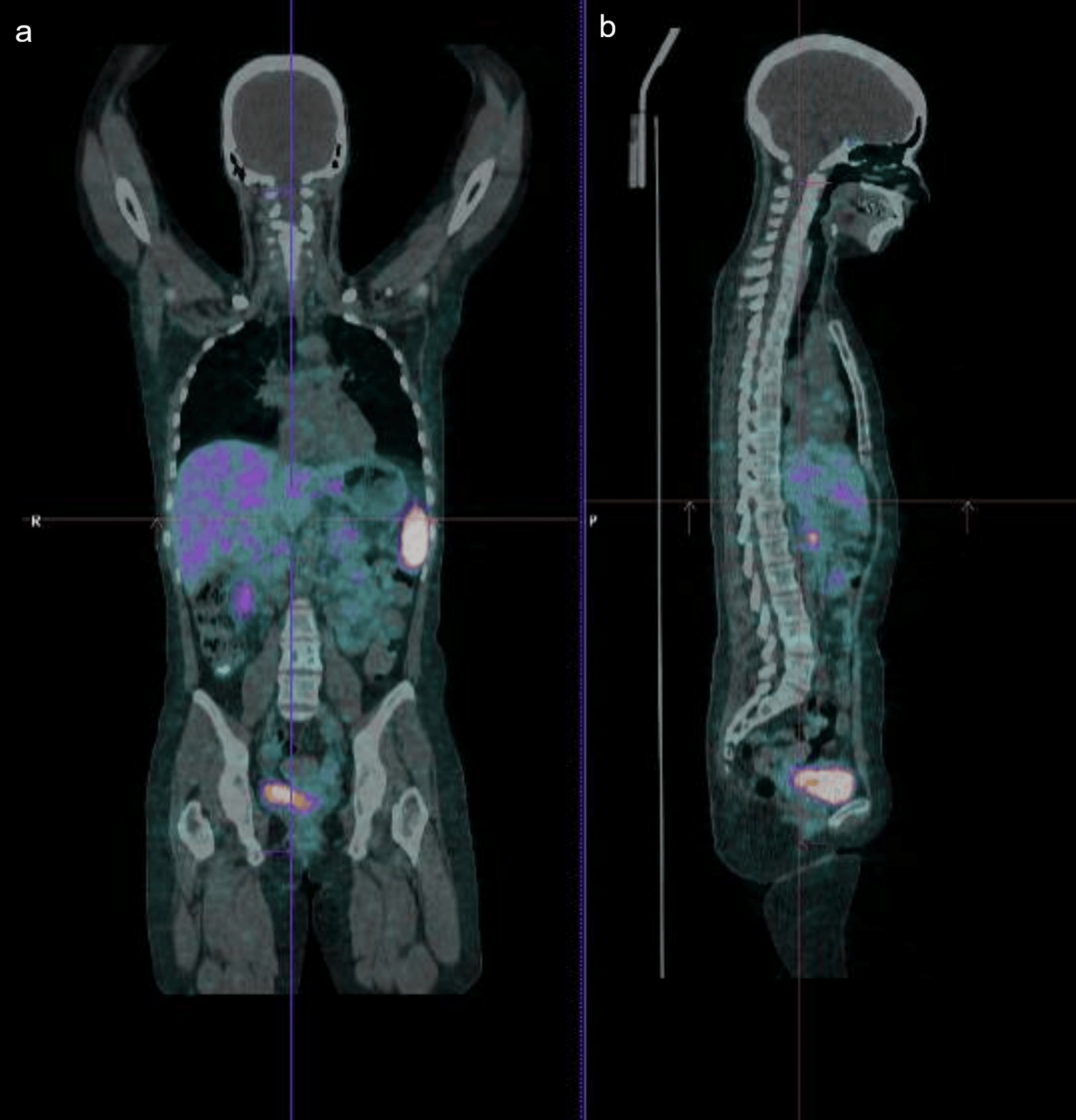
68Ga-DOTANOC PET/CT with coronal (a) and sagittal (b) views showing no evidence of lesions with increased somatostatin receptor expression. ^68^Ga-DOTANOC PET/CT: gallium-68 DOTANOC positron emission tomography.

A biopsy of the L4 vertebral lesion demonstrated a well-differentiated, low-grade NET. Immunohistochemistry showed diffuse positivity for pancytokeratin (AE1/AE3) and synaptophysin, and the proliferation index (Ki-67) was less than 2%. Surprisingly, both octreotide scintigraphy and gallium-68 DOTANOC positron emission tomography (^68^Ga-DOTANOC PET/CT) were negative for SSR overexpression (Figure 3).

A biopsy of one of the hepatic nodules was then performed, which demonstrated a well-differentiated, intermediate-grade NET, with diffuse positivity for cytokeratin 20 and synaptophysin and rare chromogranin-positive cells. The tumour cells were negative for cytokeratin 7, thyroglobulin and hepatocyte markers. The Ki-67 index was 10%.

The case was discussed at the multidisciplinary board, and a diagnosis of metastatic intermediate-grade NET was established, with the pancreas considered the most likely primary site based on imaging. Our team decided not to pursue an endoscopic ultrasound-guided biopsy, since the diagnosis of a metastatic NET had already been confirmed through biopsies of two different sites, and the patient was highly symptomatic, with an urgent need to start treatment.

The patient started first-line systemic treatment with 28-day cycles of capecitabine 750 mg/m² twice daily on D1-D14 and temozolamide 200 mg/m² once daily on D10-D14. She was referred to the Chronic Pain team for adjustment of analgesic medication and was proposed for palliative radiation therapy. A total of 20 Gy over five external beam fractions was administered to the dorsolumbar spinal segment and the left femoral head. She also started calcium supplementation and monthly administrations of intravenous zoledronic acid.

Chemotherapy was reasonably well tolerated, with manageable grade 1 thrombocytopenia and neutropenia, and no dose reductions or interruptions. However, the pain persisted despite recurrent increases in opioid dosage and an attempted trial of subcutaneous infusion with morphine and ketamine, with little success. Reassessment examinations at two months of systemic treatment detected a pathological fracture of L4, which was treated with vertebroplasty of L3-L5.

However, eight months after treatment initiation, the patient developed recurrent incapacitating pain, this time with greater intensity in the sacral region, with serious compromise of daily activities and no effective relieving strategies. Imaging studies revealed de novo enlargement of multiple mesenteric lymph nodes and several bone lesions, despite the stability of the liver metastases.

The case was re-discussed at the multidisciplinary board. A second surgical strategy for pain control was attempted, with sacroplasty, radiofrequency ablation and intraosseous steroid infiltration, with no significant improvement. Following a discussion with an expert panel, the patient started second-line treatment with everolimus at a dose of 10 mg per day. A favourable clinical response was evidenced by reduced pain, increased appetite and better functional status. Three months after treatment started, CT scans showed a partial response, with a marked reduction in hepatic lesions, abdominal lymph nodes and the pancreatic lesion, while bone lesions remained stable. Subsequent CT scans demonstrated stable disease. Treatment was reasonably well tolerated, with grade 2 stomatitis and hypertriglyceridaemia, for which she started fenofibrate 267 mg per day, with effective control. There was no evidence of everolimus-associated pneumonitis or late-onset toxicities.

After a stable period of 18 months, bone pain returned in multiple metastatic locations, with a refractory pattern despite increasing doses of opioids. The patient presented with recurrent emotional lability and addictive behaviour. No disease progression was detected. Following a Psychiatry referral, she was diagnosed with opioid dependence, anxiety and depressive disorders. A short hospital admission was proposed to proceed with opioid rotation to methadone. Successful pain control was achieved over more than seven years, allowing for a progressive methadone dose reduction (current dose: 10 mg per day).

The patient remained on daily treatment with everolimus 10 mg per day, clinically asymptomatic, working and socially active. After eight years of treatment, we requested an ^18^F-FDG PET/CT to support a potential decision to discontinue everolimus. The scan revealed two isolated lytic lesions in the right pubic bone and left sacroiliac junction, with discrete uptake (SUV 2.1-3.0). There were no new toxicities, and treatment was well tolerated, so we decided to maintain it. The ^18^F-FDG PET/CT was repeated one year earlier, with stability of these lesions. Regular follow-up continues, with blood tests every three to six months and CT scans every six months, with no evidence of disease progression.

Table 2 provides a summary of the key clinical events and their corresponding dates.

**Table 2:** Timeline of key clinical events. NET: neuroendocrine tumour, CAPTEM: capecitabine and temozolomide, FDG: fluorodeoxyglucose, PET/CT: positron emission tomography/computed tomography.

Date	Key clinical event
September 2014	Diagnosis of metastatic intermediate-grade pancreatic NET
October 2014	Started first-line CAPTEM
December 2014	Refractory lumbar pain; L4 pathological fracture
January 2015	Vertebroplasty (L3-L5)
July 2015	Disease progression (mesenteric lymph nodes, bone); severe lumbar and sacral pain
August 2015	Sacroplasty, radiofrequency ablation, and intraosseous steroid infiltration
November 2015	Started second-line everolimus
February 2016	Partial radiological response
March 2016-April 2017	Stable disease
May 2017	Refractory multifocal bone pain; opioid dependence diagnosed
June 2017	Opioid rotation to methadone
July 2017-October 2023	Stable disease; sustained pain control
November 2023	^18^F-FDG PET/CT: two residual lytic lesions; everolimus continued
June 2025	Stable findings on follow-up ^18^F-FDG PET/CT
July 2025-present	Ongoing everolimus treatment; no evidence of progression

## Discussion

This was a particularly challenging case in terms of both diagnosis and treatment, as the disease followed an aggressive clinical course despite an apparently favourable histology.

The use of radiolabelled somatostatin analogue imaging in the diagnosis and management of NETs is based on the typical overexpression of SSR on the surface of tumour cells [4]. However, in this case, histology, which was compatible with an intermediate-grade NET, was discordant with the negative results of octreotide scintigraphy and ^68^Ga-DOTANOC PET/CT. This was an unexpected finding, as the lack of SSR overexpression is more commonly found in poorly differentiated neuroendocrine carcinomas [4]. Nevertheless, there are some studies describing a subset of patients with intermediate- and high-grade metastatic gastroenteropancreatic NETs with lesions that remain undetectable on ^68^Ga-DOTANOC PET/CT, reflecting less differentiated tumour cells [7,8].

In addition, some lesions can also display increased uptake on ^18^F-FDG PET/CT, with ^18^F-FDG avidity associated with early disease progression and shorter survival, reflecting more aggressive biological behaviour [8,9]. Our patient underwent an ^18^F-FDG PET/CT, which demonstrated increased uptake in multiple bone lesions, but not in the liver or pancreatic lesions. This discordance may reflect intermetastatic tumour heterogeneity. The vertebral biopsy showed a low-grade lesion (Ki-67 <2%), whereas the liver biopsy demonstrated an intermediate-grade tumour (Ki-67 10%), suggesting biological variability across metastatic sites. Such heterogeneity has been increasingly recognised in gastroenteropancreatic NETs and may explain differences in SSR expression, ^18^F-FDG avidity and treatment response. This observation reinforces the importance of integrating pathological findings from different metastatic sites with functional imaging when making therapeutic decisions [7,9].

First-line treatment with chemotherapy for a high-volume, symptomatic intermediate-grade pancreatic NET was in line with the recommended strategy for these cases [10,11]. The combination of capecitabine and temozolomide (CAPTEM) achieved a median progression-free survival of 19 months in patients with intermediate-grade pancreatic NET, with a hazard ratio (HR) of 0.65 for disease progression or death compared with temozolomide monotherapy [12]. However, our patient had early and extensive disease progression, detected eight months after the start of chemotherapy. This outcome was worse than expected, but it is consistent with the aggressive disease biology suggested by the diagnostic findings discussed above.

The proposed second-line treatment with everolimus was based on the RADIANT-3 (RAD001 in Advanced Neuroendocrine Tumors-3) phase III trial, in which treatment of low- to intermediate-grade advanced pancreatic NET with this agent was superior to placebo, with an HR of 0.35 for disease progression or death and a median progression-free survival of 11 versus five months, respectively, mainly due to disease stabilisation [13]. The benefit observed with everolimus in this trial was independent of prior chemotherapy use [14]. In our case, the patient showed an early partial response to everolimus, with a marked reduction in visceral metastases and stability of the initially highly proliferative and symptomatic bone disease. To date, the patient has remained progression-free for 10 years while continuing everolimus, with sustained clinical benefit and acceptable quality of life.

Other reports of prolonged disease stability with everolimus monotherapy have been published. However, progression-free survival of this duration appears to be uncommon in the published literature, with previously reported cases and small clinical series generally describing progression-free intervals of less than three years [15-17].

Long-term treatment with everolimus is feasible only when toxicity remains manageable, as illustrated in this case, in which stomatitis and hypertriglyceridaemia were well controlled. In the RADIANT-3 trial, both were among the most frequently reported everolimus-related adverse events, with grade 3-4 events reported in a minority of patients [13,18].

This case also highlights the value of multidisciplinary management in a young patient presenting with a very symptomatic and incapacitating disease, mainly due to bone metastases. Bone metastases are an increasingly recognised manifestation of advanced NETs and are associated with increased morbidity and impaired quality of life [19]. In this case, an early referral to other specialities provided an integrated approach to pain relief and effective treatment of opioid use disorder.

We did not find evidence or specific recommendations concerning an ideal duration of treatment with everolimus or when it should be suspended in the absence of intolerable toxicity or disease progression. Future real-world studies may help clarify this issue and contribute to a more customised, cost-effective, safe and effective management of these patients.

## Conclusions

We presented a case of prolonged disease control and significant functional improvement with everolimus as second-line therapy in a patient with a highly symptomatic and rapidly progressing intermediate-grade metastatic pancreatic NET. Treatment decisions in metastatic pancreatic NET are complex, considering the heterogeneity in tumour biology and the possibility of discordance among clinical, radiological and pathological findings. This underscores the importance of a multidisciplinary approach to achieve individualised treatment strategies.

Despite advances in targeted therapies, the optimal sequencing beyond first-line treatment remains undefined, and high-quality comparative data are lacking. The appropriate duration of everolimus therapy is another question raised by this case and represents a relevant topic for future investigation. There is a critical need for ongoing research to refine treatment algorithms, identify predictive biomarkers and improve outcomes for patients with advanced pancreatic NETs.
